# Supplementary material for: A Comparison of Emergency Department Revisit Rates of Pediatric Patients between Pre-COVID-19 and COVID-19 Periods
Source: Children (Basel). 2022 Jul 4;9(7):1003. doi: 10.3390/children9071003 (PMC9322694; doi:10.3390/children9071003)
Supplement: Supplementary file 1 [file children-09-01003-s001.zip › children-1752695-supplementary.pdf]

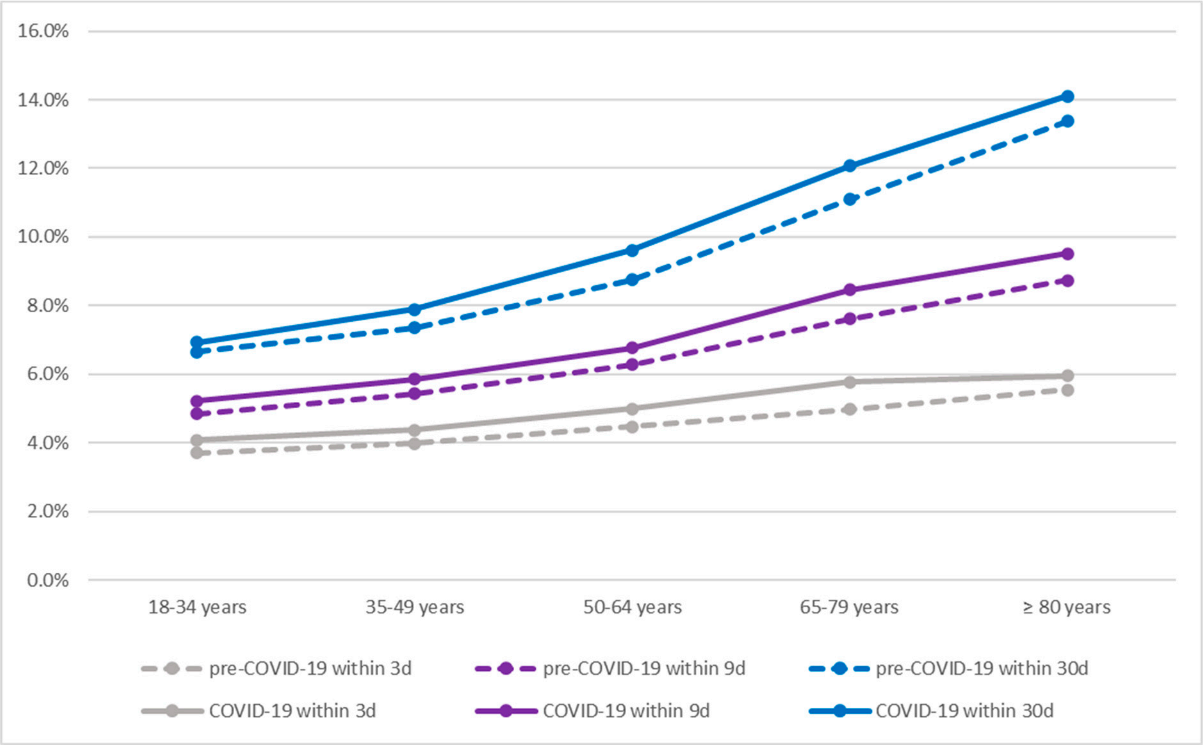

Supplemental Figure S1. Revisit rates among adults during the pre-COVID-19 and COVID-19 period based on five age-groups.
